# Procalcitonin to Reduce Antibiotic Exposure during Acute Chest Syndrome in Adult Patients with Sickle-Cell Disease

**DOI:** 10.3390/jcm9113718

**Published:** 2020-11-19

**Authors:** Keyvan Razazi, Ségolène Gendreau, Elise Cuquemelle, Mehdi Khellaf, Constance Guillaud, Bertrand Godeau, Giovanna Melica, Stéphane Moutereau, Camille Gomart, Slim Fourati, Nicolas De Prost, Guillaume Carteaux, Christian Brun-Buisson, Pablo Bartolucci, Anoosha Habibi, Armand Mekontso Dessap

**Affiliations:** 1 DHU A-TVB, Service de Médecine Intensive Réanimation, 51 Avenue du Maréchal de Lattre de Tassigny, AP-HP Hôpitaux Universitaires Henri Mondor, 94010 Créteil, France; segolene.gendreau@aphp.fr (S.G.); elise.cuquemelle@yahoo.fr (E.C.); nicolas.de-prost@aphp.fr (N.D.P.); guillaume.carteaux@aphp.fr (G.C.); Christian.Brun-Buisson@aphp.fr (C.B.-B.); armand.dessap@aphp.fr (A.M.D.); 2 IMRB, GRC CARMAS, Faculté de Santé de Créteil, Université Paris Est Créteil, 51 Avenue du Maréchal de Lattre de Tassigny, 94010 Créteil, France; 3 Service d’Accueil des Urgences, AP-HP Hôpitaux Universitaires Henri Mondor, 51 Avenue du Maréchal de Lattre de Tassigny, 94010 Créteil, France; mehdi.khellaf@aphp.fr; 4 Département d’Aval des Urgences, AP-HP Hôpitaux Universitaires Henri Mondor, 51 Avenue du Maréchal de Lattre de Tassigny, 94010 Créteil, France; constance.guillaud@aphp.fr; 5 Service de Médecine Interne, AP-HP Hôpitaux Universitaires Henri Mondor, 51 Avenue du Maréchal de Lattre de Tassigny, 94010 Créteil, France; bertrand.godeau@aphp.fr; 6 Service d’Immunologie Clinique et Maladies Infectieuses, AP-HP Hôpitaux Universitaires Henri Mondor, 51 Avenue du Maréchal de Lattre de Tassigny, 94010 Créteil, France; giovanna.melica@aphp.fr; 7 Service de Biochimie, AP-HP Hôpitaux Universitaires Henri Mondor, 51 Avenue du Maréchal de Lattre de Tassigny, 94010 Créteil, France; stephane.moutereau@aphp.fr; 8 Département de Virologie, Bactériologie, Parasitologie-Mycologie, AP-HP Hôpitaux Universitaires Henri Mondor, 51 Avenue du Maréchal de Lattre de Tassigny, 94010 Créteil, France; camille.gomart@aphp.fr (C.G.); slim.fourati@aphp.fr (S.F.); 9 Unité U955, INSERM, Université Paris Est, 94010 Créteil, France; pablo.bartolucci@aphp.fr (P.B.); anoosha.habibi@aphp.fr (A.H.); 10 French Sickle Cell Referral Center, Laboratory of Excellence GR-Ex, 51 Avenue du Maréchal de Lattre de Tassigny, AP-HP Hôpitaux Universitaires Henri Mondor, 94010 Créteil, France

**Keywords:** sickle-cell disease, acute chest syndrome, procalcitonin, antibiotic, bacterial infection

## Abstract

Acute chest syndrome (ACS) is a major complication of sickle-cell disease. Bacterial infection is one cause of ACS, so current guidelines recommend the routine use of antibiotics. We performed a prospective before–after study in medical wards and an intensive-care unit (ICU). During the control phase, clinicians were blinded to procalcitonin concentration results. We built an algorithm using the obtained measurements to hasten antibiotic cessation after three days of treatment if bacterial infection was not documented, and procalcitonin concentrations were all <0.5 μg/L. During the intervention period, the procalcitonin algorithm was suggested to physicians as a guide for antibiotic therapy. The primary endpoint was the number of days alive without antibiotics at Day 21. One-hundred patients were analyzed (103 ACS episodes, 60 in intervention phase). Possible or proven lung infection was diagnosed during 13% of all ACS episodes. The number of days alive without antibiotics at Day 21 was higher during the intervention phase: 15 [14–18] vs. 13 [13,14] days (*p* = 0.001). More patients had a short (≤3 days) antibiotic course during intervention phase: 31% vs 9% (*p* = 0.01). There was neither infection relapse nor pulmonary superinfection in the entire cohort. A procalcitonin-guided strategy to prescribe antibiotics in patients with ACS may reduce antibiotic exposure with no apparent adverse outcomes.

## 1. Introduction

Sickle-cell disease (SCD) is globally one of the most common severe monogenic disorders. Patients with SCD are at increased risk of severe bacterial infection resulting primarily from reduced or absent splenic function. Acute chest syndrome (ACS) is a major complication of SCD, and a significant cause for morbidity and mortality in adult patients [1]. ACS is characterized by fever and/or respiratory symptoms with new pulmonary infiltrates. Bacterial infection is documented in only a minority of cases [1,2]. Since establishing a definitive etiology is not always possible, empirical antibiotic therapy is often used in ACS. National guidelines and expert consensus strongly recommend antibiotic therapy for almost all patients with ACS despite the low quality of the supporting evidence [3,4,5].

Procalcitonin is currently the most useful biomarker used to distinguish sepsis from other causes of inflammation and determine the bacterial origin of a pneumonia. Its concentrations are raised during bacterial invasion rather than viral infection [6]; its elevation magnitude correlates with infection severity, and decreasing levels over time correlate with infection resolution [7]. Procalcitonin guidance was previously reported as safe to reduce duration of antibiotic treatment in critically ill patients [8] and in those with community-acquired pneumonia [9]. We conducted this study to assess whether a procalcitonin antibiotic-prescribing guideline, implemented for the treatment of ACS, would result in less exposure to antibiotics than in usual care without a higher rate of adverse events.

## 2. Experiment Section

### 2.1. Study Design

This was a single-center prospective observational before–after study of adult patients with SCD admitted for ACS to one of three medical wards or the intensive care unit of a French SCD referral center. ACS diagnosis was defined as an acute illness with fever and/or respiratory symptoms, accompanied by a new pulmonary infiltrate on a chest X-ray in a patient with genetically proven SCD. Exclusion criteria were severe immunosuppression (i.e., neutropenia, hemopathy, chemotherapy, and organ transplant), pregnancy or lactation, age less than 18 years, documented extrapulmonary infection, and antibiotics administered for more than 24 h before inclusion. The primary endpoint was the number of days alive without antibiotics at Day 21. Secondary endpoints included compliance to procalcitonin algorithm during the intervention phase, relapse, and superinfection. Good compliance to the algorithm was defined as a discontinuation of antibiotic therapy within 24 h after algorithm advice [8]. Superinfection was defined as the isolation from the same or another site of one or more pathogens different from that identified during the first infectious episode, together with clinical signs or symptoms of infection. Relapse was defined as the growth of one or more of the initial causative bacterial strains (i.e., same genus, species) from a second sample taken from the same infection site 48 h or more after stopping of antibiotics, combined with clinical signs or symptoms of infection. The following data were recorded: baseline characteristics (including type of hemoglobinopathy, steady-state hemoglobin value, history, and long-term treatment for SCD), triggering factors of ACS, documented bacterial or viral infection, treatment and outcome of ACS, and Sequential Organ Failure Assessment (SOFA) score, which quantifies organ dysfunction, and was used to adjust for patient severity. Rapidly progressive ACS was defined according to a previous publication [10].

### 2.2. Antibiotic Management

Patients received antibiotics according to the current French guidelines for ACS [5], notably in case of fever above 38 °C or signs of sepsis. Procalcitonin concentration was assessed during the first three days of ACS using the electrochemiluminescence immunoassay on automatic analyzer Cobas 6000 e601 (Elecsys BRAHMS PCT, Roche Diagnostics, Meylan, France). The lower limit of detection of the assay was 0.02 ng/mL and functional assay sensitivity was 0.06 ng/mL. During the first period (March 2011 to January 2012) of the study (control phase), clinicians were blinded to procalcitonin-concentration results. At the control phase’s end, these measurements were used to build an algorithm by a panel of SCD experts. The intervention phase began when procalcitonin measurements were available in all wards of our institution (May 2015 to April 2016). During this intervention phase, the procalcitonin algorithm was suggested to physicians as a guide for antibiotic treatment, but the final decision to start or stop antibiotics remained at their discretion. Except for procalcitonin intervention, management of ACS was similar throughout the entire study. Patients received a standardized treatment protocol for ACS in accordance with current French guidelines [5].

### 2.3. Documentation of Lung Infection

Before introducing the antibiotics, we collected blood culture, sputum for gram stain and culture, and urine for *Legionella pneumophila* serogroup 1 and *Streptococcus pneumoniae* urinary antigen tests (BinaxNOW, Portland, ME, USA). Serologic tests for *Mycoplasma pneumoniae* (ELISA-Medac^®^, Wedel, Germany), *Chlamydophila pneumoniae* (ELISA-plus Medac^®^ for IgG; ELISA-Medac^®^ for IgM, Wedel, Germany), and *Legionella pneumophila* (ELISA-Zeus Scientific^®^, Branchburg, NJ, USA; if positive, Indirect Immunofluorescence Assay IFA-Eurobio^®^, Les Ulis, France) were performed at baseline and, when possible, two weeks later. Criteria for a full microbiological documentation were as follows. *M. pneumoniae* pulmonary infection was defined by a positive serology: presence of IgM with an index > 1 and (IgG) titer > 10 AU/mL and kinetics of IgG titer of a primary infection, or IgM index < 1, or IgG titer < 10 AU/mL, but IgG emergence or increased IgG titer by a factor of three or more on a second sample two weeks or more after the first one.

*C. pneumoniae* pulmonary infection was defined by a positive serology: presence of IgM with an index > 1 and IgG titer > 25 AU/mL and kinetics of IgG titer of a primary infection, or IgM index < 1, or IgG titer < 25 AU/mL, but IgG emergence or increased IgG titer by a factor of two or more on a second sample two weeks or more after the first one.

*L. pneumophila* pulmonary infection was defined by one of the following: (i) a positive serology with high antibody titer (>256 AU/mL) by IFA at admission, (ii) an increase in antibody titer by a factor of at least four 15 days later with a second titer >128 AU/mL, (iii) a positive polymerase chain reaction (PCR) on sputum, (iv) a positive urinary antigen test for *L. pneumophila* serogroup 1, or (v) a positive culture of sputum or blood.

Viral bronchopulmonary infection was determined if any of the respiratory viruses included in the multiplex PCR [with extraction performed on QIAsymphony SP (Qiagen®, Hilden, Germany), Duplex amplification and detection of the following targets performed on Applied Biosystems Step One machine (Applied Biosystems, Foster City, CA, USA): Influenza A/B R-GENE^®^, RSV/hMPV R-GENE^®^, Rhino and EV/Cc R-GENE^®^, AdV/hBoV R-GENE^®^, BioMérieux, Marcy l’Etoile, France) was detected on a nasopharyngeal swab. High-quality sputum samples were defined by ≤10 squamous epithelial cells and ≥25 polymorphonuclear cells per field [11]. A lung infection was deemed proven, possible, or absent when one of the above-mentioned microbiological criteria was fully fulfilled, partially fulfilled, or not fulfilled, respectively.

### 2.4. Statistical Analysis

The study was designed to establish whether the procalcitonin-guided strategy was superior in terms of appropriate use of antibiotics as assessed by the number of days alive and without antibiotics at Day 21. Assuming a mean of 14 (±3) days without antibiotics for the control group, 98 patients would provide 90% power at a two-sided α = 0∙05 to detect a two-day increase in the number of days alive without antibiotics with the intervention. Continuous data were expressed as median (25th–75th percentiles) or mean ± standard deviation as appropriate and were compared using the Mann–Whitney test. Categorical variables, expressed as percentages, were evaluated using the χ^2^ test or Fisher’s exact test. Since the aim of the algorithm was to hasten antibiotic cessation, we analyzed factors associated with a short (≤3 days) antibiotic therapy course. To override the potential difference of illness severity between the two groups of patients, association between the intervention phase and a shorter antibiotic course was adjusted on the SOFA score. Statistical significance was defined as p values of less than 0.05. Data were analyzed using IBM SPSS Statistics for Windows (version 19.0, IBM Corp Armonk, NY, USA).

### 2.5. Ethics

The study was approved by the institutional ethics committee (Comité de Protection des Personnes Ile de France V) as a component of standard care, and patient consent was waived. Written and oral information about the study was given to the patients or their families.

## 3. Results

### 3.1. ACS Episodes, Lung Infections, and PCT Dosage

Among 114 screened patients, 14 had an exclusion criterion: eight had received antibiotics for more than 24 h before inclusion, five had a documented nonpulmonary infection, one was an organ-transplant recipient, and one refused data collection. One-hundred patients were included, for a total of 103 ACS episodes (43 during the control phase and 60 during the intervention phase). A total of 47 (46%) ACS cases were admitted to the intensive care or step-down unit. Baseline characteristics of patients are shown in Table S1 in the Supplementary Materials. In the entire cohort, lung infection was proven, possible, or absent in 7 (7%), 6 (6%), and 90 (87%) episodes, respectively. Pathogens involved in proven or possible infections are reported in Table 1. Clinical and biological data at diagnosis are shown in Table S2 in the Supplementary Materials. Median procalcitonin level at ACS diagnosis was 0.22 µg/L (0.11–0.6), with a maximal value during hospitalization of 0.29 µg/L (0.18–0.71). Among patients with proven bacterial infection, median PCT level was 0.21 µg/L (0.2–0.8).

Procalcitonin concentration was <0.5µg/L at Days 1–3 for 66/103 patients (64%), and higher values of procalcitonin concentrations were associated with greater severity, as shown in Table S3 in the Supplementary Materials. PCT concentration on Day 1 was 0.5 µg/L (0.2–2.9) in patients admitted to the ICU versus 0.2 µg/L (0.1–0.4) in patients admitted to medical wards (*p* = 0.001).

Considering suspected or proven bacterial infections (n = 12), we found an area under the curve (AUC) of 0.627, 95% IC (0.44–0.81) for maximal procalcitonin concentration. When only considering proven bacterial infections (n = 6), we found an AUC of 0.75, 95% IC (0.49–1.0). Sensitivity, specificity, positive predictive value, and negative predictive values for a maximal procalcitonin concentration of ≥0.5 µg/L were 0.42, 0.65, 0.14, and 0.89 for proven or probable bacterial infection, and 0.5, 0.65, 0.08, and 0.95 for proven bacterial infection, respectively.

### 3.2. Procalcitonin Algorithm for Acute Chest Syndrome

**Figure 1:**
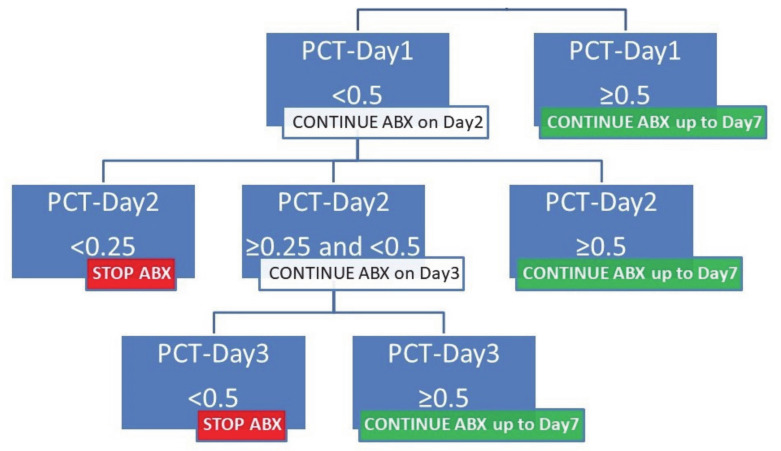
Guidelines for continuing or stopping of antibiotics according to procalcitonin concentrations in patients with acute chest syndrome. PCT, procalcitonin concentration in microg/L.

There were not enough proven or possible infections during the control phase to formally assess the diagnostic accuracy of procalcitonin. At the end of the control period, on the basis of previous studies, including the PRORATA trial [12], and on the observation that none of the proven infected patients during the control phase had consecutive procalcitonin values <0.5 μg/L during the first three days of ACS, a panel of local SCD experts proposed an algorithm to hasten antibiotic cessation after three days of treatment if bacterial infection was not documented, and serial procalcitonin concentrations were all <0.5 μg/L (Figure 1).

### 3.3. Compliance with Algorithm and Antibiotic Use

**Figure 2:**
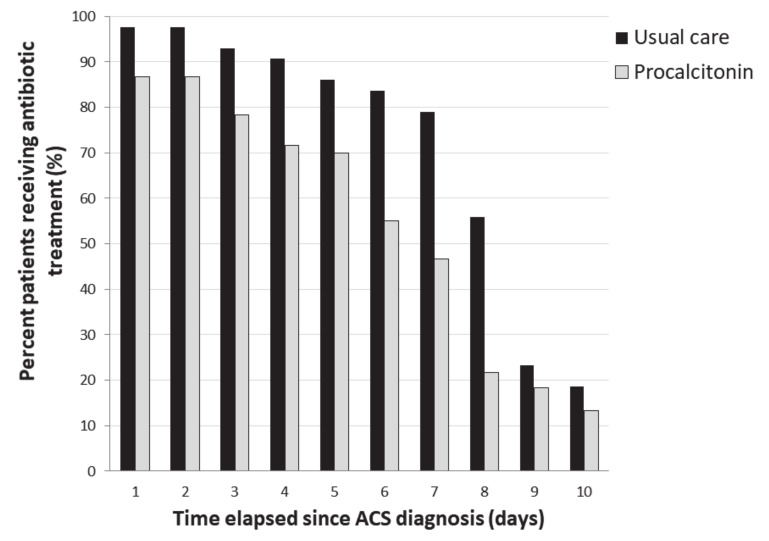
Antibiotic exposure over time in patients with acute chest syndrome according to usual care or intervention group.

During the intervention phase, 25/60 patients (42%) had an interruption of antibiotic therapy within 24 h after algorithm recommendation, 38% had a prolonged antibiotic therapy, and for 20% patients, antibiotic therapy was shorter than that recommended in the algorithm. Comparisons between patients for whom the protocol was followed and their counterparts are summarized in Table S4 in the Supplementary Materials. The number of days alive without antibiotics at Day 21 was higher during the intervention phase as compared to the control phase: 15 (14–18) vs. 13 (13–14) days (*p* = 0.001). The proportion of patients receiving antibiotics between inclusion and Day 21 was lower during the intervention phase as compared to the control phase (Figure 2). More patients received a short (≤3 days) course of antibiotics during the intervention phase as compared to the control phase: 18 (31%) vs. 4 (9%) (*p* < 0.01). The intervention phase was still associated with shorter antibiotic courses (≤3 days) after adjustment on the severity of illness as assessed by SOFA score (OR of 4.1 (1.2–13), 95% CI, *p* = 0.018).

### 3.4. Complications

There was neither infection relapse nor pulmonary superinfection in the entire cohort. One extrapulmonary superinfection occurred in each phase: peripheral intravenous catheter-related infection during the intervention phase, and bacteremia associated with bone-joint infection during the control phase. More patients required transfusions during the control phase as compared to the intervention phase (Table 2). Hospital length of stay was similar during the control and intervention phase: 7 (6–11) vs. 8 (6–12) days (*p* = 0.315). One patient died due to a noninfectious intracerebral hemorrhage.

## 4. Discussion

In this first study testing a procalcitonin-guided antibiotic treatment strategy during ACS, we observed substantially lower antibiotic exposure with this intervention.

Previous studies using different cut-off levels [13,14,15,16] with a high negative predictive value reported elevated procalcitonin as a marker of severe bacterial infection in patients with SCD and vaso-occlusive crisis. Our results are in accordance with trials performed either in emergency departments [9,17,18,19] or in primary care [20] to reduce antibiotic exposure. However, more studies looking at procalcitonin in the SCD population are needed, given the moderate accuracy of the biomarker to diagnose bacterial infection in this setting and the lack of definite cut-off value. Recent meta-analysis of individual participant data from 12 countries showed that the use of procalcitonin to guide the initiation and duration of antibiotic treatment resulted in lower antibiotic consumption, a lower risk for antibiotic-related side effects, and a lower risk of mortality [12]. Elevated procalcitonin in our study was associated with greater severity. Although there is little evidence that serum procalcitonin level at hospital admission for community-acquired pneumonia could predict mortality [21], procalcitonin indicated invasive bacterial infection in sickle-cell patients [14,22], which is a significant contributor to morbidity and mortality. Unlike previous protocols, our procalcitonin-guided antibiotic algorithm only involved the first three days, a choice driven in part by the desire to reduce blood sampling in the context of chronic anemia. Procalcitonin guidance was associated with a two-day reduction in antibiotic exposure, a result similar to that in previous published trials [12]. This reduction in antibiotic exposure may lessen antibiotic selective pressure with potential benefits in an era of increasing drug resistance. We found no difference in the length of stay in the hospital, but this outcome may be influenced by various factors not directly linked to the duration of antibiotic treatment, such as morphine requirement for associated vaso-occlusive crisis.

Several limitations of our study should be mentioned. First, our trial was monocentric, not randomized, and of limited size in a selected group of adults with ACS. Rapidly progressive ACS could not be assessed. Our findings need replication (including in a randomized controlled fashion). Our data cannot be extrapolated to other situations during SCD, for example, in patients having previously received antibiotics (in whom procalcitonin might be lower even in the presence of infection) or in case of extrapulmonary infections. Second, the sensitivities and specificities of the available microbiological diagnostic tests were imperfect, and some pathogens may have been missed. In addition, invasive procedures to obtain specimens directly from the lung were not usually performed. However, previous studies reported a low diagnostic yield of bronchoalveolar lavage in this population [2]. Our data are consistent with those of previous prospective studies suggesting that bacterial pneumonia is infrequent in adults with ACS [1,2]. Third, since there were not enough proven or possible infections during the control phase, the algorithm was built up by a panel of SCD experts according to previous studies. Compliance to the procalcitonin algorithm was low, without details as to why the algorithm was not followed in some patients. Antibiotic discontinuation occurred within 24 h after algorithm recommendation for 42% patients, and antibiotic therapy was prolonged for 38% patients. However, we were still able to show significantly reduced antibiotic use during the 21-day period after inclusion. Adherence to the procalcitonin algorithm varied among previous studies, ranging from 44% to 100%, but with similar effects in trials with high or low adherence [12]. Antibiotic stewardship is important in this population, with locally high prevalence of penicillin-resistant pneumococcal infections [23] and profound underdosing of antibiotics secondary to augmented renal clearance [24].

In conclusion, our preliminary results suggest that a procalcitonin-guided strategy may have a role in shortening the duration of antibiotic therapy with no apparent adverse outcomes during ACS in SCD. However, the use of this biomarker to predict bacterial infection in this situation should be performed with caution.

## Figures and Tables

**Table 1:** Proven and documented lung infections in adult patients with acute chest syndrome, with corresponding procalcitonin levels.

Pathogen	Proven Infections	Possible Infections	Procalcitonin Levels, µg/L
			Median, SD, (Range)
*Staphylococcus aureus*	2	4 *	Day 1: 0.73, 23.64, (0.1–59)
			Day 2: 2.38, 7.79, (0.1–16.7)
			Day 3: 0.18, 8.29, (0.1–14.5)
*Streptococcus pneumoniae*	1		Day 1: 0.2, Day 2: 0.2, Day 3: 0.2
*Enterobacter aerogenes*	1		Day 1: 0.21, Day 2: 1.07, Day 3: 75.9
*Mycoplasma pneumoniae*	1	2 #	Day 1: 0.1, Day 2: 8.88, Day 3: 3.39, 4.5, (0.2–6.57)
*Legionella pneumophila*	1		Day 1: 0.2; Day 3: 0.1
Influenza virus	1		Day 3: <0.1

* low-quality sputum samples; # first serology showed presence of IgM index > 1 and IgG = 25 AU/mL in one case, and presence of IgM index > 1 and IgG negative for the other case, without kinetics of IgG performed two weeks or more after infection onset.

**Table 2:** Evolution and outcome of 103 episodes of acute chest syndrome, and according to study period.

Parameter	All Episodes	Study Period		*p*-Value
		Control	Intervention	
	(n = 103)	(n = 43)	(n = 60)	
ACS-associated treatments				
Exchange transfusion	28 (27)	19 (44)	9 (15)	0.001
Exchange transfusion before Day 3	25 (24)	18 (42)	7 (12)	<0.001
Red-blood-cell transfusion	26 (25)	15 (35)	11 (18)	0.06
Red-blood-cell transfusion before Day 3	24 (23)	13 (30)	11 (18)	0.2
Phlebotomy	7 (7)	3 (7)	4 (7)	>0.99
Exchange and single transfusion	47 (46)	29 (67)	18 (30)	<0.001
Exchange and single transfusion < Day 3	45 (44)	29 (67)	16 (27)	<0.001
Mechanical ventilation	3 (3)	2 (5)	1 (2)	0.6
Antibiotics duration, days				
Total antibiotics duration	7 (5–8)	8 (7,8)	6 (3–7)	<0.001
Beta-lactam duration	6 (2–7)	7 (2–8)	5 (2–7)	0.05
Macrolide duration	0 (0–6)	5 (0–7)	0 (0–3)	0.001
Antibiotics-free day at Day 21, days				
Total antibiotics-free days	14 (13–16)	13 (13–14)	15 (14–18)	0.001
Beta-lactam-free days	15 (13–19)	14 (13–19)	16 (14–19)	0.05
Macrolide-free days	21 (15–21)	16 (14–21)	21 (18–21)	0.001
Three days or fewer of antibiotics	22 (22)	4 (9)	18 (30)	0.01
Microbiological diagnostic criteria	13 (13)	4 (9)	9 (15)	0.39
Proved infection	7 (7)	1 (2)	6 (10)	0.23
Partial microbiological diagnostic criteria	6 (6)	3 (3)	3 (3)	0.27
Antibiotics reintroduction	5 (5)	0 (0)	5 (8)	0.07
Infection relapse	0 (0)	0 (0)	0 (0)	
Pulmonary reinfection	0 (0)	0 (0)	0 (0)	
Extrapulmonary infection	2 (2)	1 (2)	1 (2)	>0.99
Clostridium infection	0 (0)	0 (0)	0 (0)	
ICU admission	47 (46)	25 (58)	22 (37)	0.03
Length of hospitalization, days	7.0 (6.0–12.0)	7 (5.0–11.0)	8.0 (6.0–12.0)	0.3
Rehospitalization	5 (5)	1 (2)	4 (7)	0.3
Inhospital death	1 (1)	0 (0)	1 (2)	>0.99

Data presented as median (25th–75th percentiles) or number (percentage); ACS, acute chest syndrome; ICU, intensive-care unit.

## Supplementary Materials

The following are available online at https://www.mdpi.com/2077-0383/9/11/3718/s1. Table S1: Baseline characteristics of 100 patients with acute chest syndrome according to PCT group; Table S2: Clinical and biological data at diagnosis and during hospital stay during 103 episodes of acute chest syndrome according to study period; Table S3: Characteristics of 103 episodes of acute chest syndrome according to procalcitonin concentration from Day 1 to 3; Table S4: Characteristics of the 63 episodes of acute chest syndrome included in the intervention phase according to algorithm compliance.
