# Supplementary material for: Procalcitonin to Reduce Antibiotic Exposure during Acute Chest Syndrome in Adult Patients with Sickle-Cell Disease
Source: J Clin Med. 2020 Nov 19;9(11):3718. doi: 10.3390/jcm9113718 (PMC7699579; doi:10.3390/jcm9113718)
Supplement: Supplementary file 1 [file jcm-09-03718-s001.pdf]

Online supplement

Procalcitonin to reduce exposure to antibiotics during acute chest syndrome in adult patients with sickle cell disease. PROSTA study

Keyvan Razazi[1,2], MD, Ségolène Gendreau[1,2], MD, Elise Cuquemelle[1,2], MD, Mehdi Khellaf[3], MD, Constance Guillaud[4], MD, Bertrand Godeau[5], MD, PhD, Giovanna Melica [6], MD, Stéphane Moutereau[7], Camille Gomart, [8] MD, Slim Fourati [8]MD, PhD, Nicolas de Prost[1,2,10] MD, PhD, Guillaume Carteaux[1,2,10] MD, PhD, Christian Brun-Buisson[1,2], MD,Pablo Bartolucci [9] MD, PhD, Anoosha Habibi, [9] MD, Armand Mekontso Dessap [1,2,10]MD, PhD

Table S1. Baseline characteristics of 100 patients with acute chest syndrome according to PCT-group.

Parameter	All patients (n=100)	Study period: Control (n=41)	Study period: Intervention (n=59)	*p* value
Age, years	31 [26-36]	29 [26-37]	32 [27-36]	0.7
Male gender	53 (53)	26 (63)	27 (46)	0.08
Weight, kg	65 [57-74]	65 [57-75]	64 [57-74]	0.9
Height, cm	173 [168-178]	174 [169-180]	172 [168-176]	0.2
Baseline total Hb, g/dl	9.0 [8.0-9.7]	8.5 [8.0-9.0]	9 [8.0-10.0]	0.3
SS genotype	86 (86)	36 (88)	50 (85)	0.9
**Past medical history:**				
Previous ACS	80 (80)	34 (83)	46 (78)	0.5
Previous VOC	98 (98)	40 (98)	58 (98)	>0.99
Stroke	9 (9)	3 (7)	6 (10)	0.7
Retinopathy	12 (12)	4 (10)	8 (14)	0.8
Priapism[1]	11 (21)	6 (23)	5 (19)	0.7
Bone necrosis	27 (27)	13 (32)	14 (26)	0.4
Heart disease	14 (14)	6 (15)	8 (14)	0.9
Alloimmunization	16 (16)	5 (12)	11 (19)	0.4
**Baseline treatments**				
Transfusion therapy	4 (4)	3 (5)	1 (2)	0.6
Hydroxyurea treatment	43 (43)	15 (37)	28 (48)	0.3
Home oxygen therapy	6 (6)	5 (12)	1 (2)	**0.04**
Anti-pneumococcal vaccination	53 (53)	18 (46)	35 (59)	0.2

Data are presented as median [25th-75th percentiles] or number (percentage); Hb, hemoglobin; ACS, acute chest syndrome; VOC, vaso-occlusive pain crisis.

Table S2. Clinical and biological data at diagnosis and during hospital stay during 103 episodes of acute chest syndrome according to study period.

Parameter	All episodes (n=103)	Study period Control (n=43)	Study period Intervention (n=60)	*p* value
**Type of precipitating factor**				
VOC before ACS	80 (78)	35 (81)	45 (75)	0.4
Surgery	8 (8)	1 (2)	7 (12)	0.1
Infection	2 (2)	1 (2)	1 (2)	>0.99
Other	13 (13)	6 (14)	7 (12)	0.7
**Symptoms**				
Fever (T>38°C)	72 (70)	27 (63)	45 (75)	0.2
Chest pain	90 (87)	38 (88)	57 (87)	0.8
Extrathoracic pain during ACS	75 (73)	31 (72)	44 (73)	0.9
Cough	18 (21)	7 (21)	11 (22)	>0.99
Pulmonary crackles	93 (90)	39 (91)	54 (90)	>0.99
Jugular venous distension	6 (8)	2 (7)	4 (8)	>0.99
Mean blood pressure, mm Hg	93 [82-107]	101 [91-111]	89 [79-100]	**<0.001**
Heart rate, beats/min	98 [89-110]	98 [86-112]	98 [90-110]	>0.99
Respiratory rate, breaths/min	24 [20-30]	25 [20-33]	24 [20-30]	0.2
Oxygen therapy, L/mn	3.0 [2.0-3.0]	3.0 [2.0-3.0]	3.0 [2.0-3.0]	0.5
Saturation, %	98 [95-99]	98 [96-100]	98 [94-99]	0.07
Acute cor pulmonale	9 (10)	5 (14)	4 (8)	0.5
Laboratory values at ACS diagnosis				
White cell count, 10^9/L	15.1 [10.9-18.2]	16.7 [11.3-21.2]	14.5 [9.9-17.2]	0.1
Platelet count,10^9/L	295 [211-393]	314 [212-440]	289 [210-343]	0.2
Total hemoglobin, g/dL	8.2 [6.9-9.5]	8.1 [6.3-9.5]	8.3 [7.1-9.5]	0.6
Lactate dehydrogenase, IU/L	492 [347-663]	546 [369-736]	432 [309-634]	**0.02**
Maximum C-reactive protein, mg/L	100 [40-160]	109 [62-164]	84 [38-171]	0.3
Procalcitonin at ACS diagnosis, µg/l	0.22 [0.11-0.6]	0.2 [0.15-0.46]	0.2 [0.1-0.7]	0.7

Data are presented as median [25th-75th percentiles] or number (percentage). T, temperature; ACS, acute chest syndrome; VOC, vaso-occlusive pain crisis.

Table S3. Characteristics of the 103 episodes of acute chest syndrome, according to procalcitonin level on day 1 to 3.

Parameter	All episodes (n=103)	PCT level (day 1, 2 and 3) ≥ 0.5µg/L (n=37)	PCT level (day 1, 2 and 3) < 0.5µg/L (n=66)	*p* value
**Symptoms at ACS diagnosis**				
Fever (T>38°C)	72 (70)	34 (92)	38 (58)	**<0.001**
Chest pain	90 (87)	31 (84)	59 (89)	0.5
Extrathoracic pain during ACS	75 (73)	28 (76)	47 (72)	0.6
Oxygen therapy > 3L/mn	19 (18)	10 (27)	9 (14)	0.09
**Laboratory values at ACS diagnosis**				
White cell count, 109/L	15.1 [10.9-18.2]	17.2 [13.3-23.2]	13.2 [10.4-17.0]	**0.004**
Platelet count,109/L	295 [211-393]	272 [178-349]	307 [227-415]	0.09
Total hemoglobin, g/dL	8.2 [6.9-9.5]	8.3 [7.2-9.5]	8.1 [6.9-9.5]	0.9
Lactate dehydrogenase, IU/L	492 [347-663]	619 [374-1007]	466 [326-594]	**0.003**
Maximum C-reactive protein, mg/L	100 [40-160]	174 [100-261]	81 [39-130]	**0.003**
Procalcitonin at ACS diagnosis, µg/l	0.2 [0.1-0.6]	0.7 [0.5-4.2]	0.2 [0.1-0.2]	**<0.001**
Exchange and single transfusion <day 3	45 (44)	22 (60)	23 (35)	**0.02**
Antibiotic duration	7 [5-8]	7 [6-11]	7 [3-8]	**0.007**
Invasive mechanical ventilation	3 (3)	2 (5)	1 (2)	0.3
ICU admission	47 (46)	23 (62)	24 (36)	**0.01**
SAPS II	12 [6 -18]	18 [8 -21]	10 [6 -15]	**0.009**
SOFA score	3.0 [2.0-4.0]	4.0 [2.5-5.0]	3.0 [1.8-4.0]	**0.002**
Length of hospitalization, days	7.0 [6.0-12.0]	11.0 [7.0-14.0]	7.0 [5.0-10.0]	**0.002**
Re-hospitalization	5 (5)	2 (5)	3 (5)	> 0.99
In-hospital death	1 (1)	1 (3)	0 (0)	0.4

Data are presented as median [25th-75th percentiles] or number (percentage); VOC, vaso-occlusive crisis; ACS, acute chest syndrome; ICU, intensive-care unit; SAPS II, simplified acute physiological score; SOFA, sequential organ failure assessment.

Table S4. Characteristics of the 63 episodes of acute chest syndrome, included in intervention phase, according to algorithm compliance.

Parameter	All episodes (n=60)	Antibiotic discontinuation: Earlier or according to protocol (n=37)	Antibiotic discontinuation: Prolonged duration (n=23)	*p* value
Age, years	32 [27-36]	32 [27-38]	32 [26-35]	0.9
Male gender	27 (45)	16 (43)	11 (48)	0.7
SS genotype	51 (85)	33 (89)	18 (78)	0.5
Previous ACS	47 (78)	26 (70)	21 (91)	0.1
Previous VOC	59 (98)	36 (97)	23 (100)	> 0.99
**Type of precipitating factor**				
VOC before ACS	45 (75)	25 (68)	20 (87)	0.1
Surgery	7 (12)	7 (19)	0 (0)	**0.04**
Infection	1 (2)	1 (3)	0 (0)	> 0.99
**Symptoms**				
Fever (T>38°C)	45 (75)	28 (76)	17 (74)	0.9
Chest pain	52 (87)	31 (84)	21 (91)	0.7
Extrathoracic pain during ACS	44 (73)	27 (73)	17 (74)	0.9
Cough	11 (22)	6 (20)	5 (24)	0.7
Pulmonary crackles	54 (90)	33 (89)	21 (91)	> 0.99
Mean blood pressure, mm Hg	89 [79-100]	85 [80-98]	91 [77-102]	0.7
Heart rate, beats/min	98 [90-110]	96 [82-107]	100 [91-118]	0.1
Respiratory rate, breaths/min	24 [20-30]	24 [20-28]	24 [20-38]	0.4
Oxygen therapy, L/mn	3.0 [2.0-3.0]	3 [2-4]	3 [2-3]	0.06
Oxygen therapy > 3L/mn	10 (17)	10 (27)	0 (0)	**0.009**
Saturation, %	98 [94-99]	98 [96-99]	95 [90-98]	**0.006**
Acute cor pulmonale	4 (8)	4 (12)	0 (0)	0.3
**Laboratory values at ACS diagnosis**				
White cell count, 109/L	14.5 [9.9-17.2]	15.3 [9.6-19.4]	14.4 [10.6-15.8]	0.4
Platelet count,109/L	289 [210-343]	288 [210-344]	300 [204-345]	0.9
Total hemoglobin, g/dL	8.3 [7.1-9.5]	8.0 [7.0-9.5]	8.4 [7.4-9.5]	0.4
Lactate dehydrogenase, IU/L	432 [309-634]	431 [305-625]	433 [326-656]	0.9
Maximum C-reactive protein, mg/L	84 [38-171]	101 [36-191]	57 [38-148]	0.5
Procalcitonin at ACS diagnosis, µg/l	0.2 [0.1-0.7]	0.2 [0.1-0.9]	0.2 [0.1-0.5]	0.3
Exchange and single transfusion <day 3	16 (27)	10 (27)	6 (26)	0.9
Antibiotic duration	6 [3-7]	4 [2-7]	7 [5-9]	**0.001**
3 days or less antibiotics	18 (30)	18 (49)	0 (0)	**<0.001**
Proved infection	6 (10)	2 (9)	4 (11)	> 0.99
Antibiotics reintroduction	5 (8)	1 (3)	4 (17)	0.07
Invasive mechanical ventilation	1 (2)	1 (3)	0 (0)	> 0.99
Extra-pulmonary infection	2 (3)	0 (0)	2 (9)	0.1

ICU admission	22 (37)	14 (38)	8 (35)	0.8
SAPS II	11 [6 -20]	15 [6 -21]	9 [6 -13]	0.3
SOFA score	3.0 [2.0-4.0]	3.0 [1.5-4.0]	3.0 [2.0-4.0]	0.9
Length of hospitalization, days	8.0 [6.0-12.0]	8.0 [5.5-12.0]	7.0 [6.0-12.0]	0.9
Re-hospitalization	4 (7)	0 (0)	4 (17)	**0.02**
In-hospital death	1 (2)	1 (3)	0 (0)	> 0.99

Data are presented as median [25th-75th percentiles] or number (percentage); VOC, vaso-occlusive crisis; ACS, acute chest syndrome; ICU, intensive-care unit; SAPS II, simplified acute physiological score; SOFA, sequential organ failure assessment.
